# A Rapid and Consistent Method to Identify Four SARS-CoV-2 Variants during the First Half of 2021 by RT-PCR

**DOI:** 10.3390/vaccines10030483

**Published:** 2022-03-21

**Authors:** Marco Fabiani, Katia Margiotti, Manuela Sabatino, Antonella Viola, Alvaro Mesoraca, Claudio Giorlandino

**Affiliations:** 1 ALTAMEDICA, Human Genetics, Viale Liegi 45, 00198 Rome, Italy; katia.margiotti@artemisia.it (K.M.); antonella.viola@artemisia.it (A.V.); alvaro.mesoraca@artemisia.it (A.M.); claudio.giorlandino@artemisia.it (C.G.); 2 Rome Center for Molecular Design, Department of Drug Chemistry and Technology, Sapienza University, Piazzale Aldo Moro 5, 00185 Rome, Italy; manuela.sabatino@uniroma1.it; 3 ALTAMEDICA, Fetal-Maternal Medical Centre, Department of Prenatal Diagnosis, Viale Liegi 45, 00198 Rome, Italy

**Keywords:** SARS-CoV-2, COVID-19, VOC, Delta, B.1.1.7, Alpha, TaqMan probe

## Abstract

Since 2020, the COVID-19 pandemic has spread worldwide, causing health, economic, and social distress. Containment strategies rely on rapid and consistent methodology for molecular detection and characterization. Emerging variants of concern (VOCs) are currently associated with increased infectivity and immune escape (natural defence mechanisms and vaccine). Several VOCs have been detected, including Alpha variant (B.1.1.7), Beta variant (B.1.351), Gamma variant (P.1/B.1.1.28.1) and Delta variant (B.1.617.2), first identified in the UK, South Africa, Brazil and India, respectively. Here, a rapid and low-cost technique was validated to distinguish the Alpha, Beta, Gamma, and Delta SARS-CoV-2 variants by detecting spike gene mutations using a real-time reverse transcription polymerase chain reaction methodology (RT-PCR). A total of 132 positive patients affected by coronavirus disease-19 (COVID-19) were analysed by employing RT-PCR to target single-nucleotide polymorphisms (SNPs) to screen spike protein mutations. All data were validated by the next-generation sequencing (NGS) methodology and using sequences from a public database. Among 132 COVID-19-positive samples, we were able to discriminate all of the investigated SARS-CoV-2 variants with 100% concordance when compared with the NGS method. RT-PCR -based assays for identifying circulating VOCs of SARS-CoV-2 resulted in a rapid method used to identify specific SARS-CoV-2 variants, allowing for a better survey of the spread of the virus and its transmissibility in the pandemic phase.

## 1. Introduction

Severe acute respiratory syndrome coronavirus 2 (SARS-CoV-2), the virus causing coronavirus disease-19 (COVID-19), was reported for the first time in Wuhan (Hubei Province, China) in December 2019 and became a major public health concern worldwide in less than 4 months. The outbreak of COVID-19 was inevitable and has evolved from a few affected people in Wuhan to more than 250 million people around the world (Coronavirus Research Center, https://coronavirus.jhu.edu/map.html, accessed on 1 January 2022) [1,2]. The World Health Organization (WHO) has defined some variants as variants of concerns (VOCs) because of their ability to significantly change the virus’ transmissibility or evasiveness. Recently, the WHO renamed the dominantly circulating variants with Greek letters, i.e., Alpha (α) for B.1.1.7 (U.K. variant), Beta (β) for B.1.351 (South African variant), Gamma (γ) for P.1 (Brazilian variant), and Delta (γ) for B.1.617.2 (Indian variant). Characterization from England regarding SARS-CoV-2 Alpha variants and, more recently, the Delta variant in India, have further exacerbated the pandemic situation. The Alpha variant, first identified in the south of England in September 2020, has since spread more than 30 different countries [3]. The Alpha strain has 23 novel mutations; this variant had rapidly replaced other SARS-CoV-2 lineages occurring in the region and has been established to have greater transmissibility through modelling and clinical correlation studies [4,5]. At the end of 2020, two other new variants with increased transmission and potential clinical importance emerged: the Beta variant identified in South Africa and the Gamma variant identified in Brazil [6]. The Alpha variant has several mutations located in the spike region, including the 69del, D614G, 70del 144del, E484K, S494P, N501Y, A570D, P681H, T716I, S982A, D1118H, and K1191N substitutions [7]. In Italy, on 18th March 2021, the prevalence of the Alpha England variant was 86.7%, with values ranging between 63.3% and 100% in individual regions, whereas the prevalence of the Gamma variant was 4.3% (0–36.2%), ranging between 0% and 36.2% in individual regions; at the time of the survey, the other monitored variants had a prevalence below 0.5% (www.salute.gov.it, accessed on 1 January 2022). Beta variant, located in the spike region, has the following mutations: D80A, D215G, 241del, 242del, 243del, K417N, E484K, N501Y, D614G, and the A701V substitution. The Gamma variant shares N501Y, and D614G substitutions with Alpha and Beta variants; moreover, located in the spike region, it has the following mutations: E484K, L18F, T20N, P26S, D138Y, R190S, K417T, H655Y, and the T1027I mutation [7]. The new VOC, the Delta variant, was first detected in India in December 2020 [8]. As of October 2021, the variant had been detected in over 98 countries around the world and became the dominant variant in most of those countries, including the UK, Israel, and Italy. The Delta variant, located in the spike region, has the following mutations: T19R, V70F, T95I, G142D, E156-, F157-, R158G, A222V, W258L, K417N, L452R, T478K, D614G, P681R, and D950N [7]. Multiple mutations in the spike protein and other important genomic areas are common in these variants. Alpha, Beta and Gamma SARS-CoV-2 lineages share the N501Y mutation, which is of interest among the other mutations because it is involved in the receptor-binding mechanism and has been identified to increase the binding affinity to human angiotensin-converting enzyme 2 (ACE 2) [8]. In addition, Gamma, Beta, and Delta variants carry the E484K mutation in the spike protein’s receptor-binding domain (RBD). E484K variants were previously reported to escape neutralizing antibodies [9]. On 26 November 2021, the WHO proposed SARS-CoV-2 variant B.1.1.529, named Omicron, as a fifth VOC. Concern about the Omicron variant was primarily raised due to its large number of mutations in the genome, especially in its S gene [10]. There are several mutations, including H69_V70del, K417N, S477N, T478K, E484A, and N501Y, that have been previously identified from the other VOCs [11]. Because VOC lineages contain mutations enabling SARS-CoV-2 virus to spread more quickly in people and more easily evade natural or vaccine-induced immunity, it is important to monitor their prevalence to better understand the evolution ofSARS-Co-2. SARS-CoV-2 lineages are characterized by sequencing-based technologies, and increased monitoring of variants’ spread is necessary to develop more rapid and lower-cost methods compared to the NGS platform. In this study, an RT-PCR test was developed for rapid identification of Alpha, Beta, Gamma, and Delta variants in SARS-CoV-2-positive samples.

## 2. Materials and Methods

### 2.1. Sample Collection

From 1 February 2021 until 30 June 2021, Nasopharyngeal/Oropharyngeal swabs were collected in viral transport medium and processed at Altamedica laboratory (Rome) for the detection of SARS-CoV-2 virus using approved RT-PCR kits (KHB, Diagnostic kit for SARS-CoV-2, Shanghai, China). 

Among these, 132 viral RNA samples tested positive for SARS-CoV-2 and were selected for this study. The study was conducted with the consent of all participants and was approved by the internal Ethics Committee of Altamedica Laboratories, Artemisia S.p.A.

### 2.2. Detection of SARS-CoV-2 Virus by Real-Time Polymerase Chain Reaction (RT-PCR)

Detection of SARS-CoV-2 RNA was performed by RT-PCR (KHB, Diagnostic kit for SARS-CoV-2) in accordance with the manufacturer’s instructions. The assay targets three genes of SARS-CoV-2 (N, E, and ORF1ab). All samples included in the study had a cycle threshold (Ct) value of the gene targets of less than or equal to 30 for all genes.

### 2.3. RNA Extraction and cDNA Synthesis

Viral RNA was extracted from nasopharyngeal swab using a PANA 9600s automated systems extractor according to the manufacturer’s instructions and stored at −80 °C until use. A SuperScript 2 VILO cDNA synthesis Kit (Thermo Fisher Scientific, Waltham, MA, USA) was used to reverse-transcribe (RT) the SARS-CoV-2 RNA with the following protocol: 4 µL of 5× VILO™ reaction mix, 2 µL of 10× SuperScript™ enzyme mix, viral RNA (10 ng), and nuclease-free water to a final volume of 20 µL. A retrotranscriptase reaction was performed in a thermal cycler with the following program: 25 °C for 10 min, 42 °C for 60 min, and 85 °C for 5 min. To ensure enough cDNA content for NGS workflow, RNA was quantified with a Qubit 3.0 fluorometer (Thermo Fisher Scientific, Waltham, MA, USA). 

### 2.4. TaqMan RT-PCR Assay

SARS-CoV-2 mutations were determined using TaqMan^®^ Universal PCR Master Mix and a specific custom TaqMan probe. Each RT-PCR mixture consisted of 10 µL TaqMan Universal PCR Master Mix, 1 µL of TaqMan probe 10×, 1 µL cDNA template, and 8 µL of nuclease-free water. All RT-PCR assays were performed on a QuantStudio 12K Flex Real-Time (Thermo Fisher Scientific, Waltham, MA, USA). The thermal protocol parameters are reported in Table 1.

### 2.5. SNP Genotyping Panel Design for SARS-CoV-2 Variants

The trimmed SARS-CoV-2 genome sequences were downloaded from the COVID-19 Genomics UK (COG-UK) consortium website (https://www.cogconsortium.uk/data/, accessed on 1 January 2022) and from NCBI SARS-CoV-2 Resources (https://www.ncbi.nlm.nih.gov/sars-cov-2/, accessed on 1 January 2022). A genotyping panel probe was specifically designed to target spike (S) protein mutation characteristics of each SARS-CoV-2 variant of concern, as reported on PANGO lineage (https://cov-lineages.org/index.html, accessed on 1 January 2022). As shown in Table 2, the following substitutions were selected: Alpha: N501Y, H69_V70del, Y145del, A570D, P681H, T716I, S982A, D1118H; Beta: D614G, N501Y, E484K, D215G, and A701V; Gamma: D614G, N501Y, E484K, K417T, D138Y, and T1027I; Delta: D614G, T19R, L452R, and T478K. Using a custom TaqMan assay design tool, sequence-specific probes were designed to detect the target sequence region (Thermo Fisher Scientific, Waltham, MA, USA). Each assay included two TaqMan minor groove binder (MGB) probes with nonfluorescent quenchers (NFQ): a VIC dye-labelled probe to detect the reference sequence and a FAM dye-labelled probe to detect the mutation sequence in the spike (S) gene. 

### 2.6. Criteria for SARS-CoV-2 Variant Definitions

As criteria for SARS-CoV-2 variant characterization considered the presence of D614G mutation as first filter criterion, as the second step, the presence of N501Y mutations was used to distinguish the Alpha, Beta, and Gamma variants from the Delta variant. H69_V70del, Y145del, A570D, P681H, T716I, S982A, and D1118H SNPs indicated Alpha variant; D215G, E484K, and A701V indicated Beta; K417T, E484K, D138Y, and T1027I indicated Gamma variant; and T19R, L452R, and T478K indicated Delta variant.

### 2.7. Library Preparation and Sequencing

For each sample, a target amplification reaction was set up using 10 µL of cDNA, 4.5 µL of 5× Ion AmpliSeq™ HiFi mix, and 3.5 µL of water; this mixture was split into two different tubes, and 2 µL of each of the 5× ion AmpliSeq™ Primer Pool 1 and 2 were added to the corresponding tubes. Reaction of amplification was performed in a thermal cycler with the following program: 98 °C for 2 min, followed by 16 cycles at 98 °C for 15 s and 60 °C for 4 min. The previous reactions were then combined, and 2 µL FuPa reagent (Thermo Fisher Scientific, Waltham, MA, USA) was added to partially digest the primers; afterward, the mixture was incubated in a thermal cycler with the following program: 50 °C for 10 min, 55 °C for 10 min, and 60 °C for 20 min. Then, 2 µL of diluted Ion Xpress™ barcode adapters, together with 4 µL of switch solution and 2 µL DNA ligase were added to ligate the adapters to the amplified products, and the samples were incubated with the following program: 22 °C for 30 min, 68 °C for 5 min, and 72 °C for 5 min. After ligation, each DNA library was purified with magnetic beads (Agencourt™ AMPure™ XP Reagent, Beckman Coulter) and then amplified with 50 µL of Platinum™ PCR SuperMix HiFi and 2 µL of library-amplification primer mix under the following conditions: 2 min at 98 °C, 5 cycles of 15 s at 98 °C, and 1 min at 64 °C. The amplified libraries were again purified with magnetic beads, and the final concentration of each barcoded cDNA library was determined with a Qubit 3.0 fluorometer (Thermo Fisher Scientific, Waltham, MA, USA) following the manufacturer’s recommendations. Barcoded libraries were diluted to 33 pM, pooled in equal volume aliquots, and then loaded onto an Ion Chef™ instrument (Thermo Fisher Scientific, Waltham, MA, USA) for emulsion RT-PCR, enrichment, and loading into the Ion S5 Prime System (Thermo Fisher Scientific, Waltham, MA, USA). Samples were pooled together (ranging from 20 to 32) and sequenced on an Ion 530 chip (Thermo Fisher Scientific, Waltham, MA, USA). Torrent Suite™ software was used to compare base calls, read alignments, and variant calling. Reads were aligned with the Wuhan-Hu-1 NCBI Reference Genome in Torrent Suite v. 5.10.1. The following plugins were used: Coverage Analysis (v5.10.0.3), Variant Caller (v.5.10.1.19), and COVID19 AnnotateSnpEff (v.1.0.0), a plugin specifically developed for Sars-Cov-2 that can predict the effect of a base substitution. Integrative Genomic Viewer v.2.8.0 (IGV) was used to visualize the TVC (torrent variant caller) bam files to check the consistency of nucleotide calls [12].

### 2.8. SARS-CoV-2 Variant and Mutation Characterization

Raw sequence reads were aligned to the complete genome of SARS-CoV-2 Wuhan-Hu-1 isolate (Genbank accession number: NC_045512.2) and classified using the Pangolin COVID-19 Lineage Assigner tool v2.0.7 (github.com/cov-lineages/pangolin, accessed on 1 January 2022). Mutations of external datasets were defined by uploading the FASTA sequences to the NextClade website (https://clades.nextstrain.org/, accessed on 1 January 2022).

### 2.9. External Validation Dataset

All SARS-CoV-2 genome sequences were retrieved from NCBI Virus (https://www.ncbi.nlm.nih.gov/labs/virus/vssi/#/, accessed on 1 January 2022). The criteria for download were the presence of all complete genome sequences and all submitted Italian sequences. A total of 162 sequences met these criteria. The FASTA file sequences were submitted to Pangolin tools for variant calling and to the NextClade website for mutation calling.

### 2.10. Statistical Analysis

Standard statistical analyses (averages and standard deviations) were performed using Microsoft Excel and GraphPad Prism 8.4.3 for Windows. Visualization graphs in this paper were generated using Matplotlib in Python.

## 3. Results

### 3.1. Genotyping Clinical SARS-CoV-2 Samples

**Figure 1:**
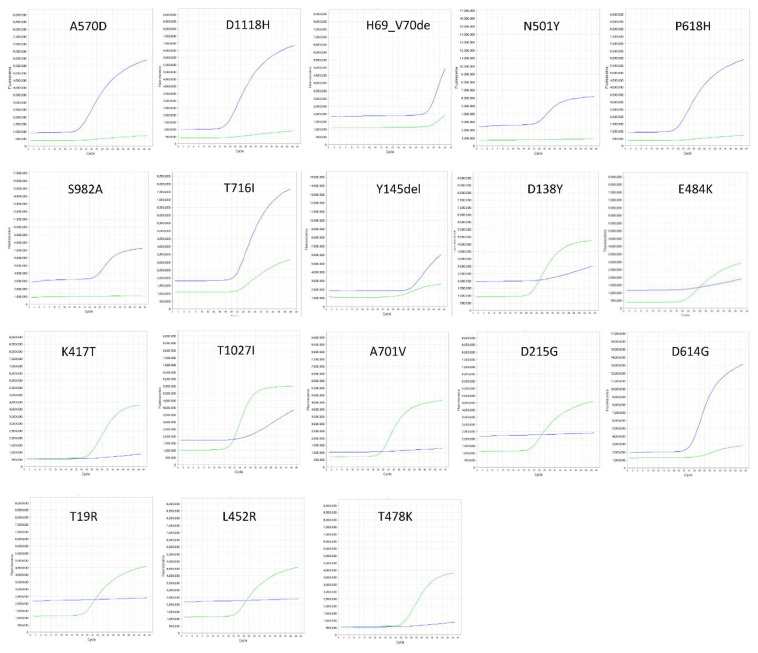
RT-PCR plots for SARS-CoV-2 Alpha variant identification. A sigmoid blue (FAM) curve represents a mutated sequence, whereas a sigmoid green (VIC) curve represents a wild-type sequence (referenced to as a Wuhan sequence).

To characterize SARS-CoV-2 variants, we genotyped 132 SARS-CoV-2-positive swab samples by RT-PCR analysis using TaqMan probes. As shown in Figure 1 and Table 2, we designed a D614G probe common to Alpha, Beta, Gamma, and Delta variants, then designed N501Y to easily distinguish Alpha, Beta, and Gamma from the Delta variant. Whereas seven probes were designed for the Alpha variant, four probes were designed for Gamma, three probes for Beta, and three probes for the Delta variant (Table 1).

Results of SARS-CoV-2 variants after RT-PCR analysis were compared with NGS sequenced data of all 132 SARS-CoV-2-positive samples and, after Pangolin classification (github.com/cov-lineages/pangolin), showed a concordance of 100% (95% CI, 97.2–100%) between NGS sequenced data and RT-PCR data for the Alpha, Beta, Gamma, Delta, and other variants not investigated (B.1.177, B.1.258, B.1.545) (Table 3). Considering each specific variant separately, the revealed concordance was 100% (61/61; 95% CI 94.1–100%) for the Alpha variant, 100% (12/12; 95% CI 73.5–100%) for the Gamma variant, 100% (5/5; 95% CI 47.8–100%) for the Beta variant, 100% (32/32; 95% CI 89.1–100%) for the Delta variant (Table 3); and 100% (22/22; 95% CI 84.6–100%) excluding the four VOCs examined in this work. The analysis of mutations using the designed TaqMan probes introduced no false positives (0/132) or false negatives (0/132). The mean technical concordance for each TaqMan assay probe, A570D, D1118H, H69_V70del, N501Y, P681H, S982A, T716I, D614G, Y145del, D138Y, E484K, K417T, T1027I, A701V, D215G, L452R, T19R, and T478K, was 97.7% (ranging from 94.7% to 99.2%).

### 3.2. Comparison between External Dataset and SNP Genotyping Panel

A total of 162 FASTA file sequences were downloaded from NCBI and submitted to Pangolin tools for variant calling. Pangolin submission identified 34 Alpha variants, 9 Gamma variants, 6 Beta variants, 40 Delta variants, 13 A variants, 2 B variants, and 58 B.1 variants with other sub-lineages (different from examined VOCs). Afterward, the 162 FASTA file sequences were submitted to the NextClade website for SNP calling, and all data were compared with our designed genotyping panel. The designed genotyping panel correctly classified Alpha, Beta, Gamma, and Delta variants downloaded from the external dataset. Moreover, using the D418G probe, the B.1 lineage was discriminated from A and B lineage in 100% of cases (82/82), as all viral sequences in the external database with D418G mutations belong to the B.1 head lineage (Table S1). 

### 3.3. Frequency and Informativity of the SNP Marker for SARS-CoV-2 Variant Characterization by RT-PCR Probe

To determine the technical and biological efficiency of each TaqMan probe assay to discriminate between SARS-CoV-2 variants, we considered the frequency and the informativity of the single-nucleotide polymorphism markers. Informativity was defined as the percentage of the difference between the frequency of a single SNP in one SARS-CoV-2 variant with respect to its frequency in all other SARS-CoV-2 variants (Table 4). In Table 4, we show the frequency and informativity of the single-nucleotide marker for each variant group.

We found that the three most informative SNPs for the Alpha variant are D1118H, S982A, and T716I with, 100%, 99%, and 99% informativity, respectively (Table 4); the two most informative mutations for the Gamma variant are K417T and D138Y, with 92% and 92% informativity, respectively; the two most informative mutations for the Beta variant are D215G and A701V, with 100% and 99% informativity, respectively; the two most informative mutations for the Delta variant are L452R and T478K, with 100% and 97% informativity, respectively (Table 4).

## 4. Discussion

The emergence of SARS-CoV-2 variants with the potential for increased transmission, disease severity, and resistance to vaccine-induced immunity is a grave concern. A simple screening assay to monitor the emergence and spread of these strains, despite expensive and time-consuming sequencing of the virus genome, may help implement public health strategies to counter these and future strains. PCR-based assays that produce results in a few hours can be a useful method for early detection and prevalence calculation of VOCs (i.e., Alpha, Beta, Gamma, and Delta). TaqMan assays have been widely used for genotyping due to their low cost, high sensitivity, and tolerance of variation in the quality and quantity of input DNA. Several groups are currently developing and evaluating such techniques [13,14]. In this study, we developed a genotyping panel assay using specific *RT-PCR* custom probes able to distinguish the main SARS-CoV-2 variants with 100% concordance with respect to the gold-standard NGS technology (Table 3). The developed custom genotyping panel probes differentiate between the Alpha, Beta, Gamma, and Delta, as well as all B.1-derived variants (all carrying the D614G mutation), from the A.1 (Wuhan) lineage. To determine the capacity of SNPs to discriminate SARS-CoV-2 variants, the informativity of each selected SNP was calculated. As shown in Table 4, the informativity of individual SNPs ranged from 85% to 100%. Bioinformatics analysis of 82 SARS-CoV-2 genomes downloaded from NCBI suggested that our SNP genotyping panel correctly assigned SARS-CoV-2 without the need to sequence all SARS-CoV-2 genomes. All selected SNPs are S-protein mutations of concern selected from PANGO lineage reports (https://cov-lineages.org/index.html (accessed on 27 November 2021)). Substitution D614G is common to Alpha, Beta, Gamma, and Delta SARS-CoV-2 variants; N501Y is common to Alpha, Beta, and Gamma SARS-CoV-2 variants; and E484K is common to Beta, Gamma, and Delta variants. All of these recently characterized mutations are known to enhance the transmissibility of the affected virus [15,16]. Among the 132 sequenced clinical samples was an already-reported B.1.525 variant; at the time that this variant was reported on PANGO lineage, its frequency in Italy was less than 0.9% (Table S2). Interestingly, Beta shares the Y145del mutation with the Alpha variant and shares the E484K mutation with Beta and Gamma. Additional studies about mutations of B.1.525 variants are required to implement additional mutations to discriminate this variant from other variants with *RT-PCR* TaqMan probes. 

Moreover, a new SARS-CoV-2 VOC emerged during the drafting of this manuscript: Omicron [17]. The first sequenced Omicron case was reported in Botswana on 11 November 2021, and a few days later, another sequenced case was reported in Hong Kong in a subject traveling from South Africa [18]. Now, SARS-CoV-2 variants are screened by NGS methodology in positive samples and several other valid genotyping methods, such as the double-mismatch, allele-specific RT-PCR [19], Sanger-based approach [20], as well as high-resolution melting analysis [21,22]. Double-mismatch, allele-specific RT-PCR methodology requires two parallel RT-PCR reactions to detect any SARS-CoV-2 sequence other than the target variant of concern. The primers and probes are custom-synthesized. Garson et al. claim that these double-mismatch, allele-specific RT-PCR tests show a strong and reliable correlation with results obtained through genome sequencing [19]. Bezerra et al. used a Sanger sequencing methodology and reported that they were able to identify and discriminate all known SARS-CoV-2 variants by sequencing a single PCR fragment [20]. Finally, Aoki A et al. were able to identify six different types of SARS-CoV-2 viral clade by high-resolution melting (HRM) analysis [21,22].

In this work, a nasopharyngeal swab of positive patients was used as starting material. Although many authors have shown that detecting Sars-CoV-2 is possible even in saliva samples [23], in this study, we chose a nasopharyngeal swab considering that it remains the gold standard in most clinical settings worldwide.

A limit of our methodology to take into account is that all samples included in the study had a cycle threshold (Ct) value less than or equal to 30 for all analysed genes. This implies that samples with a Ct greater than 30 could lead to a lack of amplification of one or more mutations, causing a Sars-CoV-2 variant misclassification.

Our results show that *RT-PCR* methodology using TaqMan probes directed to specific SNPs can be a useful and rapid genotyping tool for SARS-CoV-2-positive samples at a low cost, although it cannot detect new mutations. Testing laboratories may also consider designing their own genotyping panels based on regional or national datasets to maximize the virus survey. The robustness of our panel is further increased by the use of two probes in the same reaction, one complementary to the WT sequence and one complementary to the mutant sequence. All diagnostic structures qualified to detect SARS-CoV-2 in an oropharyngeal swab with this low-cost technique could further screen positive SARS-CoV-2 samples to identify variants, thereby providing valuable genomic data to investigate outbreaks, potentially identifying transmission pathways, linking local and regional cases and helping to inform possible interventions. Notably, efficient methods for tracking the transmission of certain lineages could be vital in situations where mutations are associated with increased transmission or severity of disease or vaccine failure. A limitation of this study is related to the limited number of samples, especially for the P.1 variant, counting only six positive patients, and for the B.1.351 variant, counting only one sample. To overcome this problem, we used an external sequenced dataset to confirm the validity of our results. 

## 5. Conclusions

Targeting specific SARS-CoV-2 mutations by *RT-PCR* methodology using TaqMan probes is a rapid and consistent method used to identify specific SARS-CoV-2 variants. Based on the reached informativity results (ranging from 85% to 100%), herein, we showed that using such custom TaqMan probes is recommend for efficient differentiation of the four investigated variants. When used on a large scale by a wide number of private or public laboratories, this method could be helpful to better survey the spread of virus variants in the pandemic phase. 

## Figures and Tables

**Table 1:** Thermal protocol used for TaqMan RT-PCR assay.

Stage	Temperature	Time
UNG incubation	50 °C	2 min
Polymarase activation	95 °C	10 min
PCR (40 cycles)	95 °C	15 s
	60 °C	1 min

**Table 2:** Analysed mutations of spike protein present in Alpha, Beta, Gamma, and Delta SARS-CoV-2 variants.

	Sars-Cov-2 VOC			
Mutation	Alpha	Beta	Gamma	Delta
A570D	Present	-	-	-
D1118H	Present	-	-	-
H69_V70del	Present	-	-	-
N501Y	Present	Present	Present	-
P681H	Present	-	-	-
S982A	Present	-	-	-
T716I	Present	-	-	-
Y145del	Present	-	-	-
D138Y	-	-	Present	-
E484K	-	Present	Present	-
K417T	-	-	Present	-
T1027I	-	-	Present	-
A701V	-	Present	-	-
D215G	-	Present	-	-
D614G	Present	Present	Present	Present
L452R	-	-	-	Present
T19R	-	-	-	Present
T478K	-	-	-	Present

**Table 3:** Concordance of results of SARS-CoV-2 variant characterization between TaqMan probe *RT-PCR* and NGS sequencing.

Lineage	N	Concordance, *RT-PCR* vs. NGS
Alpha (B.1.1.7)	61/61	100% (95% CI 94.1–100%)
Beta (B.1.351)	5/5	100%(95% CI 47.8–100%)
Gamma (P.1)	12/12	100% (95% CI 73.5–100%)
Delta (B.1.617.2)	32/32	100% (95% CI 84.6–100%)
B.1 with D614G	22/22	100% (95% CI 84.6–100%)

**Table 4:** Frequency and informativity of 18 selected SNPs in 132 SARS-CoV-2 genomes.

	Frequency (%)					Informativity * (%)				
	Other Variants	Alpha	Gamma	Beta	Delta	Other Variants	Alpha	Gamma	Beta	Delta
A570D	0	98%	0	0	0	0	98%	0	0	0
D1118H	0	100%	0	0	0	0	100%	0	0	0
H69_V70del	0	97%	0	0	0	0	97%	0	0	0
N501Y	0	94%	92%	100%	0	0	0	0	0	0
P681H	5%	100%	0	0	10%	0	85%	0	0	0
S982A	0	99%	0	0	0	0	99%	0	0	0
T716I	0	99%	0	0	0	0	99%	0	0	0
Y145del	5%	94%	0	0	0	0	89%	0	0	0
D138Y	0	0	92%	0	0	0	0	92%	0	0
E484K	5%	0	100%	100%	0	0	0	0	0	0
K417T	0	0	92%	0	0	0	0	92%	0	0
T1027I	5%	0	92%	0	0	0	0	87%	0	0
A701V	0	1%	0	100%	0	0	0	0	99%	0
D215G	0	0	0	100%	0	0	0	0	100%	0
D614G	100%	99%	92%	100%	97%	0	0	0	0	0
L452R	0	0	0	0	100%	0	0	0	0	100%
T19R	0	0	0	0	91%	0	0	0	0	91%
T478K	0	0	0	0	97%	0	0	0	0	97%

* % informativity VOC = [% frequency VOC − (% frequency other VOCs + % frequency other variants)].

## Data Availability

The data that support the findings of this study are available from the corresponding author upon reasonable request.

## Supplementary Materials

The following supporting information can be downloaded at https://www.mdpi.com/article/10.3390/vaccines10030483/s1: Table S1: Presence (MUT) or absence (WT) of the specific mutation in SARS-CoV-2 genome sequences in an external dataset downloaded from NCBI Virus https://www.ncbi.nlm.nih.gov/labs/virus/vssi/#/; Table S2: Presence (MUT) or absence (WT) of the specific mutation in SARS-CoV-2 clinical samples by NGS and *RT-PCR* methods.
